# 2443: Investigating markers of early traumatic brain injury (iMet): An interim analysis

**DOI:** 10.1017/cts.2017.276

**Published:** 2018-05-10

**Authors:** Tara Rhine, Ting Sa, Nanhua Zhang, Shari Wade, Rachel P. Berger

## Abstract

OBJECTIVES/SPECIFIC AIMS: Analyze data from the first 30 children enrolled in a prospective cohort study evaluating the ability of specific serum biomarkers to distinguish children with traumatic brain injuries (TBI) from children with orthopedic injuries (OI). METHODS/STUDY POPULATION: Children ages 0<5 years were eligible if they presented to the emergency department within 6 hours of injury. Children were identified as having a TBI if they sustained a head injury and were found to have an acute injury on head CT. Children were identified as having an OI if they sustained a musculoskeletal injury significant enough to necessitate radiography per clinical care. Individual (eg, age) and clinical (eg, radiography findings) factors, as well as serum biomarkers [eg, ubiquitin C-terminal hydrolase L1 (UCH-L1), glial fibrillary acidic protein (GFAP)] were collected at time of enrollment. TBI and OI groups were compared using Wilcoxon rank-sum and Kruskal-Wallis tests. RESULTS/ANTICIPATED RESULTS: This cohort consisted of 13 children with TBI (7 with isolated skull fractures, 1 with intracranial injury, and 5 with both a skull fracture and an intracranial injury) and 17 with OI (12 with fractures). Most patients were male (67%) and White (67%), and this did not differ between groups (*p*>0.1). Children with TBI were significantly younger than children with OI, with an average (±standard deviation) age of 15±13 and 39±13 months, respectively (*p*<0.01). There was not a significant difference in time from injury to biomarker collection between TBI and OI patients at 4.1±1.8 and 5.8±2.6 hours, respectively (*p*=0.07). Median (IQR) levels of GFAP were significantly higher (*p*<0.01) in children with TBI, relative to children with OI: 220 (67–421) pg/mL Versus 37 (25–74) pg/mL, respectively. Median (IQR) levels of UCH-L1 were also significantly higher (*p*<0.01) in the TBI group, relative to children with OI: 444 (377–449) pg/mL Versus 248 (140–417) pg/mL, respectively. In a subanalysis comparing median biomarker levels across three study groups (ie, TBI with an isolated skull fracture, TBI with an intracranial injury, and OI), group differences remained significant for both biomarkers with TBI patients having higher levels, relative to OI patients, of both GFAP (*p*<0.01) and UCH-L1 (*p*=0.02). DISCUSSION/SIGNIFICANCE OF IMPACT: GFAP and UCH-L1 hold promise to improve the diagnosis of TBI in very young children. Identification of a marker of TBI that can be done in the acute care setting would advance the diagnosis of TBI in very young children, a vulnerable population for whom identification of neurological symptoms can be challenging.

